# In-City Rain Mapping from Commercial Microwave Links—Challenges and Opportunities

**DOI:** 10.3390/s23104653

**Published:** 2023-05-11

**Authors:** Roy Janco, Jonatan Ostrometzky, Hagit Messer

**Affiliations:** School of Electrical Engineering, Tel Aviv University, Tel Aviv 6997801, Israel; messer@eng.tau.ac.il

**Keywords:** ISAC, opportunistic ISAC, opportunistic sensing, smart city, environmental monitoring, RNN

## Abstract

Obtaining accurate rainfall measurements is highly important in urban areas, having a significant impact on different aspects in city life. Opportunistic rainfall sensing utilizing measurements collected by existing microwave and mmWave-based wireless networks has been researched in the last two decades and can be considered as an opportunistic integrated sensing and communication (ISAC) approach. In this paper, we compare two methods that utilize received signal level (RSL) measurements obtained by an existing smart-city wireless network deployed in the city of Rehovot, Israel, for rain estimation. The first method is a model-based approach using the RSL measurements from short links, in which two design parameters are calibrated empirically. This method is combined with a known wet/dry classification method, which is based on the rolling standard deviation of the RSL. The second method is a data-driven approach, based on a recurrent neural network (RNN), which is trained to estimate rainfall and classify wet/dry periods. We compare the results of rainfall classification and estimation from both methods and show that the data-driven approach slightly outperforms the empirical model and that the improvement is most significant for light rainfall events. Furthermore, we apply both methods to construct high-resolution 2D maps of accumulated rainfall in the city of Rehovot. The ground-level rainfall maps constructed over the city area are compared for the first time with weather radar rainfall maps obtained from the Israeli Meteorological Service (IMS). The rain maps generated by the smart-city network are found to be in agreement with the average rainfall depth obtained from the radar, demonstrating the potential of using existing smart-city networks as a source for constructing 2D high-resolution rainfall maps.

## 1. Introduction

Accurate and robust rainfall measurements are highly important for urban water management, flood risk mitigation, urban planning, transportation management, agriculture and more.

Flash floods can be caused by excessive rainfall and are characterized by rapid onset. The presence of large impervious areas, buildings and blocking of storm-water flow impose high risks of flash floods in urban areas. Flash floods are frequently responsible for loss of lives and damage to infrastructure. Climate change and increasing urbanization implies more frequent and severe flash floods in the future. Over the last decade, flash-flood forecast lead-time has expanded up to six hours due to improved rainfall forecasts; yet, unknown future precipitation remains the largest source of uncertainty of flash-flood forecasts [1]. Javier et al. [2] showed that high-resolution rainfall rate fields can provide important elements of site-specific flash-flood forecasting systems in small urban watersheds. They also commented that errors in rainfall fields produce the largest sources of uncertainty in quantitative flash-flood forecasting.

The absorption and scattering of electromagnetic waves from hydrometeors along the path of propagation cause attenuation to transmitted signals. This fact was utilized for the first time by Messer et al. [3] in 2006 for environmental monitoring from commercial microwave link (CML) signal level measurements. There, the authors suggested the use of existing cellular networks as they provide built-in monitoring facilities and are widely distributed and, hence, can serve as a high-resolution atmospheric observation network, without the required cost of constructing new designated environmental sensing networks.

The rapid growth of urbanization and population, in addition to advancements in communication technology, have promoted the concept of smart cities, which support city operations intelligently with minimal human interaction [4]. As smart cities become larger and the number of everyday objects connecting to existing networks is rising, the number of wireless communication links is rising, as well, to support the increase in demand.

Smart cities provide local municipalities with broadband networks using optical fibers and/or millimeter wave links to support high-bandwidth communication. These networks provide infrastructure for wireless WIFI network solutions, communication of public and education buildings, connection to security cameras and more. Signals transmitted in the E-band frequency range (60 GHz to 90 GHz) provide high bandwidth but do not penetrate easily through buildings and can be attenuated by foliage and rain to a large extent [5].

Due to these limitation, E-band links are usually used for short-range communication. These short links are also common in the wireless backhaul of emerging 5G cellular networks. Such networks can have dense coverage over urban areas, which brings up great opportunities to obtain high-resolution rainfall maps.

Similar to the use of CMLs from cellular backhaul networks to serve as meteorological sensors, smart-city wireless links can be exploited, as well, to provide opportunistic meteorological sensing abilities from dense urban areas without the additional cost of placing dedicated rain measuring devices.

The network hardware reports the CMLs’ transmitted signal level (TSL) of the transmitter and the received signal level (RSL) of the receiver, which are usually logged by network operators for network management purposes (or can be accessed directly using the simple network management protocol (SNMP)). The attenuation of the transmitted signal is, therefore, obtained by:

(1) A(t)=TSL(t)−RSL(t),

where A(t), in dB, is referred to as the total attenuation and *t* indicates time.

As mentioned before, the transmitted signal is scattered by hydrometeors along the path of propagation, which constitutes the rain-induced attenuation of the signal. The total attenuation of a signal is modeled as a summation of free-space attenuation (“path loss”), gaseous attenuation, rain induced attenuation and more.

To summarize, total attenuation A(t) can be described by the simplified model of (2):

(2) $$A\left(t\right)=A_{r}\left(t\right)+A_{WA}\left(t\right)+A_{BL}+N\left(t\right),$$

where $A_{r}\left(t\right)$ is rainfall-induced attenuation, $A_{WA}\left(t\right)$ is attenuation due to the wet antenna effect [6] and $A_{BL}\left(t\right)$ is attenuation caused by sources other than rain, such as free-space attenuation and gas attenuation, and is referred to as the baseline attenuation. Usually, $A_{BL}\left(t\right)$ slowly changes with time compared to rain-induced factors. N(t) is an additive measurement noise assumed independent among all links.

Attenuation due to rain is usually modeled using the power law [7]:

(3) $$A_{r}\left(t\right)=aR^{b}\left(t\right)L$$

where a,b are coefficients depending on the link-specific frequency, antenna polarization and rain drop size distribution [8]; *L*, in km, is the link path length; and R(t), in mm/h, is the rain rate (i.e., the rain intensity).

A comprehensive study on rainfall and water vapor sensing with microwave links operating at E-band frequencies was conducted in [9]. The authors showed that E-band links are more sensitive to rain than links operating in the K-band (and, specifically, the 15–40 GHz range) and can observe light rainfall. However, those links were shown to be affected more by errors related to the rainfall drop size distribution.

It was shown in [10] that the power-law equation from (3) is less accurate for short links. The authors also showed how recurrent neural networks (RNNs) can be used to estimate the rainfall rate from measurements recorded by a cellular network management system. The RNN-based method outperformed the traditional power-law-based method in terms of the root mean square error (RMSE).

In our previous work [11], we presented an empirical model for short links, which can be seen as a modification to the power law. We showed that rainfall estimation from short links suffers from overestimation using the power law, and the proposed model is able to eliminate the overestimation.

In this paper, we compare two methods of rainfall estimation from the attenuation measurements of short E-band links: the first is a two-parameter empirical model from [11], which corrects the attenuation measurements of short links before applying the power law. The model parameters are calibrated using attenuation measurements from a long link in the vicinity of the network. The second is a variation of a data-driven method based on a gated recurrent unit (GRU) from [12], which showed improved performance in terms of RMSE compared to the traditional algorithm based on the power law.

The main contributions of this paper are:

1. We present a comparison between an empirical short links model and an RNN-based data-driven approach for rainfall estimation from RSL measurements. We show that although the RNN-based approach performs better in terms of RMSE, the simple short links model yields similar results for moderate and strong rain intensities (higher than 5 mm/h), despite being much simpler.
2. We create high-resolution 2D maps of 24 h-accumulated rainfall, which are constructed from the estimates of either method. The constructed maps are compared against rainfall maps provided by the IMS weather radar, and both show good agreement with the ground truth.

The rest of this paper is organized as follows: Section 2 describes the data used from the city of Rehovot, Israel. In Section 3, the details of the two rainfall estimation methods are provided. In Section 4, the experimental results from the two methods are shown, and a comparison of the constructed rainfall maps to the weather radar maps is provided. Section 5 concludes this paper.

## 2. Data

RSL measurements from the smart-city network of Rehovot are recorded regularly by the network operator company SMBIT LTD (SMBIT. Ltd, Mazkeret Batya, Israel. https://www.smbit.co.il). The network consists of 66 links, where each link contains 2 sub-links for the two opposite directions. All links operate in the E-Band frequency range, namely, in the range of 70 GHz to 84 GHz, while the majority of links operate at 74.375 GHz. The RSL values are sampled every 30 s with a quantization level of 1 dB. The TSL is not recorded and is assumed to be constant.

**Figure 1:**
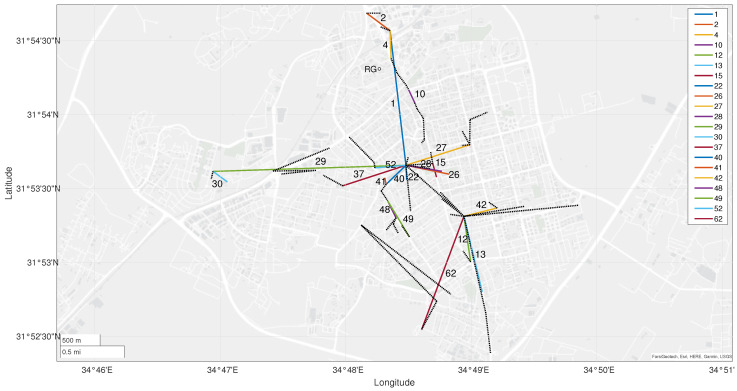
Rehovot links map. The network contains 66 links, where the longest one is link number 29, with a length of 2.42 km. Colored lines with ID numbers represent links that were used to create the rainfall maps (different colors are used for visibility purpose only). Dashed black lines represent excluded links. A rain gauge is placed in the north part of the city and is marked with a circle labeled “RG”.

Figure 1 depicts the link map of Rehovot. Colored lines represent links that were used to construct rainfall maps and are marked with an ID number. Dashed black lines represent links that had been excluded in this work due to high unrelated fluctuations of the signals levels that were detected during dry periods, as well as very short links that did not attenuate above a minimal degree. The municipality building is located in the center of the map. The majority of the antennas are located at street level, connecting traffic lights cameras. Others are placed on building rooftops, connecting schools and others to the main municipality building.

A rain gauge (the measurements are provided by The Robert H Smith Faculty of Agriculture, Food and Environment (Rehovot), The Hebrew University of Jerusalem. http://www.meteo-tech.co.il/faculty/faculty_periodical.asp?client=1 (accessed on 11 April 2023)) in the northern part of the city is used for validation and marked as ‘RG’ in Figure 1. The rain-gauge measurements are collected by the faculty of agriculture, food and environment (Rehovot), the Hebrew University of Jerusalem. It measures the accumulated rainfall for 10 min intervals, with a resolution of 0.1 mm.

### 2.1. Data Pre-Processing

**Figure 2:**
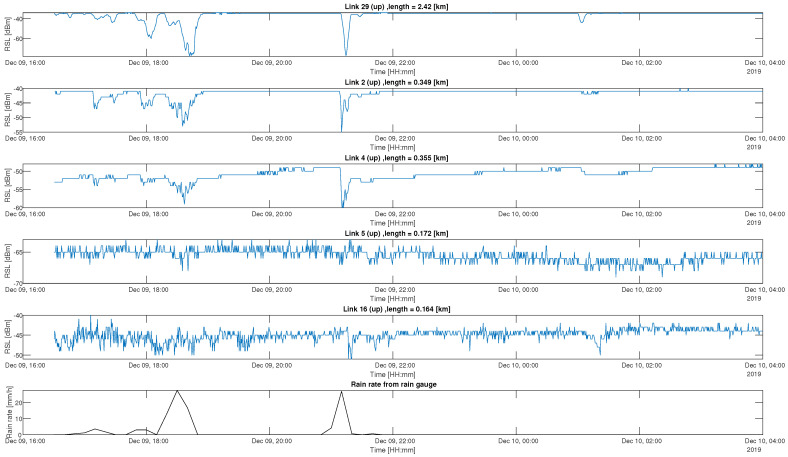
Example of time series of the raw data: The top 5 panels depict the RSL measurements, as recorded by selected links, in dBm, as a function of the time; the bottom panel represents the rain intensity, in mm/h, as was recorded by the RG as a function of the time. All panels are aligned and depicted for the same 12 h period on 9 December 2019.

Examples of RSL time-series raw data from links 29, 2, 4, 5 and 16 are shown in the top five panels of Figure 2, according to this order. The bottom panel presents the rainfall intensity measured by the rain gauge as a function of time. The RSL signals from links 29, 2 and 4 show an agreement with the rain-gauge measurements. The RSL is attenuated during rainfall, and it is approximately constant when it stops raining. Note that the RSL of link 4 rises slowly after the rainfall stopped. Links 5 and 16 show a higher noise level. These large fluctuations can stem from multi-path propagation, where the signal can be reflected from buildings, cars, vegetation or other reflective surfaces along the path of propagation [13]. Moreover, the aging of the electronics of the transmitter and the receiver can increase noise levels. Estimating rainfall from them is a much harder task and will result in large errors.

We noticed some changes in the RSL properties of some links at different years, such as different baseline levels and even changes in the range of attenuation values due to rain. Some links showed more than a 10 dB difference between the median of the winter RSL of 2021 and the winter RSL of 2020. Furthermore, links that were too short showed only small attenuation values of the RSL during rainfall, and others were not attenuated at all. We excluded those links, as well as links showing large fluctuations during dry periods. The excluded links are shown as dashed lines in Figure 1.

### 2.2. Dataset Split

The full dataset, consisting of RSL measurements from a fixed number of links is split into three datasets. The first one is used for training the network (TRAIN). The second one is used for validation and hyper-parameter tuning (VALIDATION). The last one is used for testing only (TEST).

The TRAIN set consists of data from different rain events that occurred during 1 October 2019 to 31 March 2020.

The VALIDATION set consists of data from different rain events that occurred during 1 November 2020 to 31 December 2020.

The TEST set consists of data from different rain events that occurred during 1 January 2021 to 28 February 2021.

The datasets’ durations are summarized in Table 1.

### 2.3. Data Imbalance

Since, most of the time, the weather in Israel is not rainy, the datasets are imbalanced toward dry periods. The wet and dry samples ratio are summarized in Table 2 for the different datasets.

**Figure 3:**
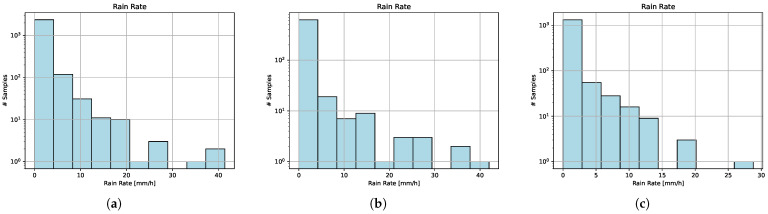
Dataset rainfall histograms in a logarithmic scale. All three datasets are imbalanced toward dry samples and light rainfall. (**a**) TRAIN dataset rain histogram. (**b**) VALIDATION dataset rain histogram. (**c**) TEST dataset rain histogram.

The wet samples are also imbalanced toward light rainfall, as can be seen in Figure 3a–c.

## 3. Rainfall Estimation

In this section, we compare the two methods of estimating the path-averaged rainfall rate. The first method we use is the empirical short links model from [11], which uses the attenuation measurements of a long link in the network as a reference for estimating the model parameters. The second method we use is a data-driven method, based on the two-step network from [12]. The main block of the network is a GRU, which is used to learn long-term dependencies of the RSL measurements.

### 3.1. Short Links Empirical Model

The empirical short links model from [11] is used to correct the observed attenuation of short links. Since the TSL is unknown, Equation (1) can not be used in order to calculate the observed attenuation. Instead, we calculate the “resting” level of the RSL, which we denote using z(t), by applying a moving median with a centered window of one week on the RSL averaged over 15 min intervals, as was conducted in [9]. The observed attenuation is, therefore, calculated by:

(4) A(t)=max(z(t)−RSL(t),0)

The model has two parameters (unique per link), which are used to correct the short-links-related inaccuracies. The model is defined as follows:

(5) $$A_{r}^{i}\left(t\right)=\frac{max(A^{i}\left(t\right)-b^{i},0)}{W^{i}}$$

where $A^{i}\left(t\right)$ is the observed attenuation, in dB, at time index *t*, of link *i*. $b^{i}$ is a parameter to compensate for a constant wet antenna attenuation and $W^{i}$ is a correction factor for short links. $A_{r}^{i}\left(t\right)$ is referred to as the rain-induced attenuation of link *i*.

The model parameters are estimated by minimizing the following cost function:

(6) $${\hat{\theta }}^{i}=\underset{\theta^{i}}{\mathrm{argmin}}{∥\frac{A_{r}^{i}\left(\theta^{i}\right)}{L^{i}}-\frac{A_{long}}{L_{long}}∥}_{2}^{2}$$

where $A_{r}^{i}$ is a vector of rain attenuation measurements from link *i*, $A_{long}$ is a vector of the observed attenuation measurements of the long link (link 29 in this case), which is used as a reference. $L^{i}$ and $L_{long}$ are the lengths of link *i* and the long link, respectively.

The wet/dry classification method from [14] is used to detect rainy periods. The method applies a rolling standard deviation (RSD) of a fixed-size window to the RSL measurements. The choice of a small window size enables for capturing more details about the variability in the signal caused by rain, at the expense of a less powerful ability to detect constant rainfall events, since the RSD will be low. The input, $RSL_{n}$, is classified as wet if the standard deviation at the given time step exceeds a threshold σ. The threshold, σ, is set for each link individually by the 80th percentile of the 30 min rolling standard deviation in the corresponding dataset, multiplied by a scaling factor. The scale factor was adopted from the work of [15] and was also used in [16]. A scaling factor of 1.2 and a 30 min window size were selected to maximize the F1 score on the VALIDATION dataset.

In the next sections, we use the short links model and the RSD method together for wet/dry classification and rainfall estimation and refer to it as the “SLM-RSD” model, which stands for short links model—rolling standard deviation.

### 3.2. Data-Driven Approach

The input of the network includes both the RSL measurements and the corresponding length and frequency of each link. The RSL measurements are sampled every 30 s, whereas the rain-gauge measurements are sampled every 10 min. A single input to the network at time-step *n* is defined as follows:

(7) $$x_{n}={\left[{\bar{\mathrm{RSL}}}_{n-(w-1)},...{\bar{\mathrm{RSL}}}_{n-1},{\bar{\mathrm{RSL}}}_{n},L,F\right]}^{T},$$

where ${\bar{\mathrm{RSL}}}_{n}$ is the normalized RSL measurement at time-step *n*; *w* is a fixed window size, which is the ratio between the sampling rate of the rain gauge and the sampling rate of the RSL; *L* is the link’s path length; and *F* is the link’s frequency. In our case, *w* is set to 20 since the rain gauge is measured every 10 min, whereas the RSL is sampled every 30 s. In this way, $x_{n}\in R^{22}$.

The RSL measurements of each link are normalized by subtracting the median value of the entire RSL time series for the given link:

(8) $${\bar{\mathrm{RSL}}}_{n}={\mathrm{RSL}}_{n}-\mathrm{median}\left({\left\{{\mathrm{RSL}}_{i}\right\}}_{i=1}^{N_{s}}\right)$$

The network is trained to classify an input sequence of non-overlapping vectors containing samples of the form (7) to a sequence of wet/dry estimates of the same length and to estimate the path averaged rainfall rate corresponding sequence. The interval between each time-step matches the rain gauge sampling rate, i.e., 10 min. For an input sequence of length *L* (representing 10·L minutes of data), the input sequence to the network will be:

(9) $${\left\{x\right\}}_{n-(L-1)}^{n}=\left\{x_{n-w(L-1)},\cdots ,x_{n-2w},x_{n-w},x_{n}\right\}$$

and the corresponding rain-gauge measurement sequence is:

(10) $${\left\{y\right\}}_{n-(L-1)}^{n}=\left\{y_{n-(L-1)},\cdots ,y_{n-2},y_{n-1},y_{n}\right\}$$

#### 3.2.1. Architecture

**Figure 4:**
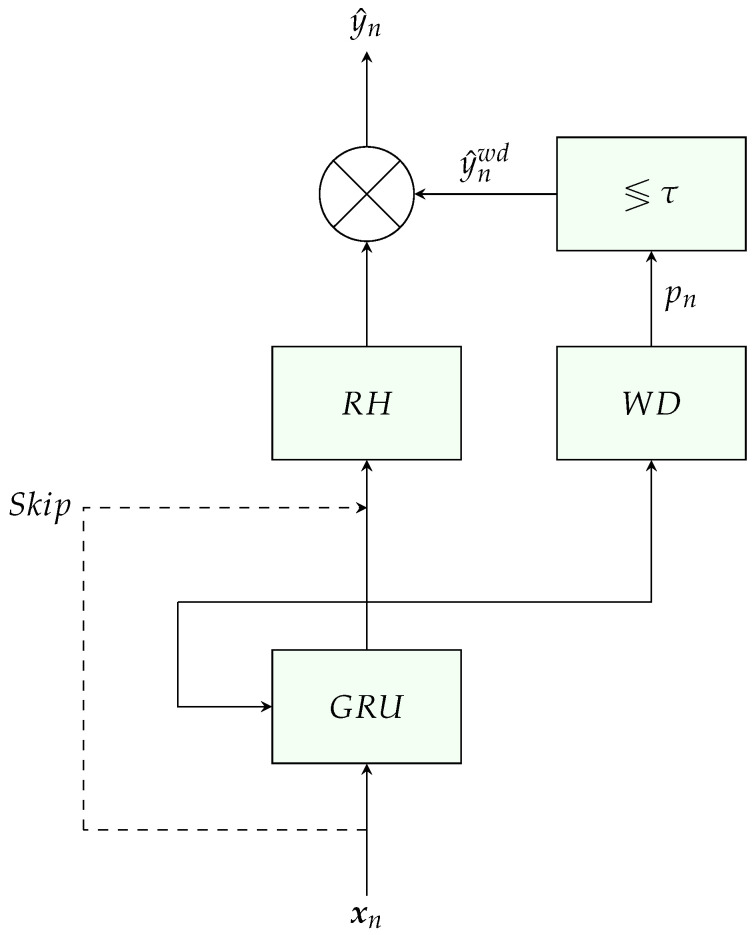
Network architecture for the estimation problem.

The architecture of the combined rain estimation and classification network is depicted in Figure 4. It is based on the two-step architecture from [12]. The network consists of three main blocks: a GRU, which is used to learn long-term dependencies between the RSL samples of the input; a rain head (RH), which converts the GRU output to rainfall estimation; and a wet/dry (WD) block, which converts the GRU output to a scalar between 0 and 1, denoted by $p_{n}$, representing the probability that the input sample, $x_{n}$, is considered wet. The probabilities are passed through a fixed threshold τ, where an output of 0 represents dry, and an output of 1 represents wet, as described below:

(11) $${\hat{y}}_{n}^{wd}=\left\{\begin{array}{cc} 1 & \mathrm{if}\;p_{n}\geq \tau \\ 0 & \mathrm{if}\;p_{n}<\tau \end{array}\right.$$

The selection of τ is conducted using a grid search on the VALIDATION dataset. Finally, the wet/dry indicator is multiplied with the RH output to produce the rain estimate, ${\hat{y}}_{n}$.

The RH consists of fully connected (FC) layers followed by ReLU activation, and the WD block consists of one layer of FC followed by sigmoid activation. A skip connection, which concatenates the input, $x_{n}$, and the GRU output as the RH input, is added.

Four configurations were tested, where we changed the RH size and also enabled the skip connection, which concatenates the network’s input to the GRU output before applying the rain head. The different configurations are described in Table 3.

#### 3.2.2. Loss Function

The loss function includes a regression term, which is a mean squared error (MSE) loss, and a classification term, which applies focal loss [17] to the wet/dry probabilities of the wet/dry outputs. Focal loss is an extension to the binary cross entropy (BCE) loss such that it reduces the weights assigned to the well-classified examples. For an output sequence ${\left\{{\hat{y}}_{n,i}\right\}}_{n=1}^{N_{s}}$ of link *i* and ground truth measurements ${\left\{y_{n}\right\}}_{n=1}^{N_{s}}$, the loss is defined as follows:

(12) $$Loss=L_{\mathrm{MSE}}+\lambda_{FL}L_{FL},$$

where

(13) $$L_{\mathrm{MSE}}=\sum_{i=1}^{N_{l}}\sum_{n=1}^{N_{s}}{\left(y_{n,i}-{\hat{y}}_{n,i}\right)}^{2},$$

(14) $$L_{FL}=\sum_{i=1}^{N_{l}}\sum_{n=1}^{N_{s}}-\alpha_{n,i}^{t}{\left(1-p_{n,i}^{t}\right)}^{\gamma }log\left(p_{n,i}^{t}\right),$$

(15) $$p_{n,i}^{t}=\left\{\begin{array}{cc} p_{n,i} & \mathrm{if}\;y_{n,i}=1\\ 1-p_{n,i} & \mathrm{if}\;y_{n,i}=0 \end{array}\right.,$$

(16) $$\alpha_{n,i}^{t}=\left\{\begin{array}{cc} \alpha & \mathrm{if}\;y_{n,i}=1\\ 1-\alpha & \mathrm{if}\;y_{n,i}=0 \end{array}\right.,$$

where $N_{s}$ is the number of samples in the sequence and $N_{l}$ is the number of links. γ and α are the focal loss hyper-parameters and $\lambda_{FL}$ is a hyper-parameter controlling the balance between the two loss terms.

#### 3.2.3. Augmentation

The data were augmented by letting the input sequences start at a random point at the beginning of each epoch. The random starting point is selected uniformly from $\left[0,N_{s}-1\right]$. This augmentation is applied on the dataset used for training only.

### 3.3. Validation Metrics

To evaluate the classification results, we use true positives (TPs), false positives (FPs), true negatives (TNs) and false negatives (FNs) as the basis for other useful classification metrics.

The first metric we used is the overall accuracy, which is defined as:

(17) $$\mathrm{Accuracy}=\frac{\mathrm{correct}\;\mathrm{predictions}}{\mathrm{total}\;\mathrm{number}\;\mathrm{of}\;\mathrm{samples}}=\frac{\mathrm{TP}+\mathrm{TN}}{\mathrm{TP}+\mathrm{FP}+\mathrm{TN}+\mathrm{FN}}$$

Since the data are imbalanced toward dry samples, and since the more challenging task is identifying wet periods, precision (18) and recall (19) were used to evaluate the model under the positive class. The F1 score (20) is the harmonic mean of the precision and recall and is useful since it summarizes both metrics into one number:

(18) $$\mathrm{Precision}=\frac{\mathrm{TP}}{\mathrm{TP}+\mathrm{FP}}$$

(19) $$\mathrm{Recall}=\frac{\mathrm{TP}}{\mathrm{TP}+\mathrm{FN}}$$

(20) $$F1=2\frac{\mathrm{Precision}\cdot \mathrm{Recall}}{\mathrm{Precision}+\mathrm{Recall}}$$

The balanced accuracy is also used for the same reasons, where it is defined by the average of the true positive rate and the true negative rate.

(21) $$\mathrm{Balanced}\;\mathrm{Accuracy}=\frac{1}{2}\left(\frac{\mathrm{TP}}{\mathrm{TP}+\mathrm{FN}}+\frac{\mathrm{TN}}{\mathrm{TN}+\mathrm{FP}}\right)$$

To validate the rainfall estimation results, the normalized bias (NBIAS) and normalized RMSE (NRMSE) are used:

(22) $$\mathrm{NBIAS}\left({\hat{R}}^{i},R_{rg}\right)=\frac{\frac{1}{N}\sum_{n}\left({\hat{R}}_{n}^{i}-R_{n}^{rg}\right)}{{\bar{R}}^{rg}}$$

(23) $$\mathrm{NRMSE}\left({\hat{R}}^{i},R_{rg}\right)=\frac{\sqrt{\frac{1}{N}\sum_{n}{\left({\hat{R}}_{n}^{i}-R_{n}^{rg}\right)}^{2}}}{{\bar{R}}^{rg}}$$

where ${\bar{R}}^{rg}$ is the average rain rate measured by the rain gauge and N is the number of samples.

## 4. Experimental Results

### 4.1. Rain Estimation and Classification

A GRU with 2 layers and a hidden layer with 256 neurons were used. The model was trained with sequence lengths of 8 h, which is equivalent to the length of 48 samples. ADAM optimizer [18] was used for training the model with a learning rate of 0.0001. The optimizer and the learning rate were empirically selected as they provided better results on the VALIDATION dataset. To avoid overfitting the training data, a dropout of probability 0.5 was applied to the RNN layers. In addition, an L2 regularization term was added to the loss function with a factor of 0.0001. The focal loss parameters were set to γ=2, α=0.95 and $\lambda_{FL}=100$. The model was trained on links 1, 2, 3, 4, 8, 10, 11 and 29.

We compared the performances of the different architectures on the TEST dataset. The results are summarized in Table 4. The threshold that maximized the F1 score on the VALIDATION dataset is presented. The BIAS and RMSE were calculated for samples from the wet class only.

The four configurations produce similar results in terms of the classification metrics. All configurations perform better than the SLM-RSD model in terms of RMSE and F1 score. From now on, we continue the results section with the “Large RH + Skip” configuration and refer to it as, simply, “RNN”.

**Figure 5:**
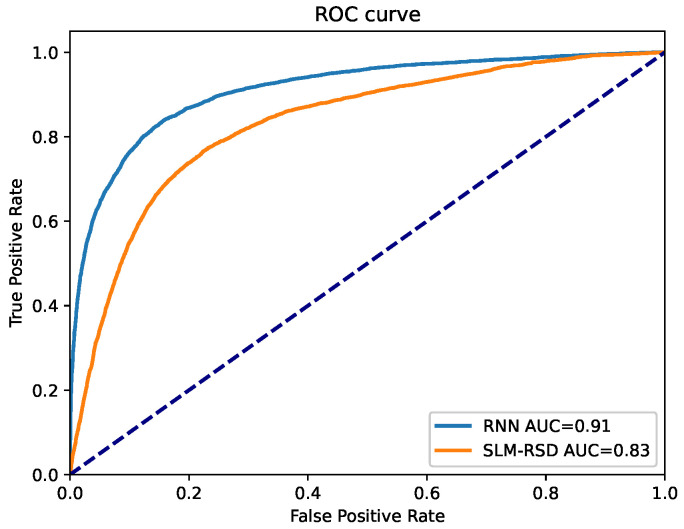
ROC curve on TEST dataset. The blue line represents the ROC curve obtained by the RNN model, achieving AUC of 0.91. The orange line represents the ROC curve obtained by the RSD method which has been used in the short links model, achieving AUC of 0.83. The dashed line represents an ROC curve of a random classifier.

We compared the receiver operating characteristic (ROC) curves and their respective area under curve (AUC) of the RNN models to the SLM-RSD model on the TEST dataset in Figure 5. The blue line represents the ROC curve obtained by the RNN model, while the orange line represents the ROC curve obtained by the RSD method. The dashed line represents an ROC curve of a random classifier. The RNN models show an improvement of around 10% in AUC compared to the RSD method. The different configurations of the RNN models show a very similar pattern to the ROC curves and yield approximately the same AUC.

The performance of both rainfall estimation methods are depicted in Table 5, where each row describes the results for each link.

Inspecting the performance of individual links, the short links model performed slightly better on links 1, 2, 22 and 40, while the RNN model performed better on the rest. Links 26 and 28 exhibit overestimation using the short links model, which can be explained by a non-optimal *W* parameter for these specific links.

**Figure 6:**
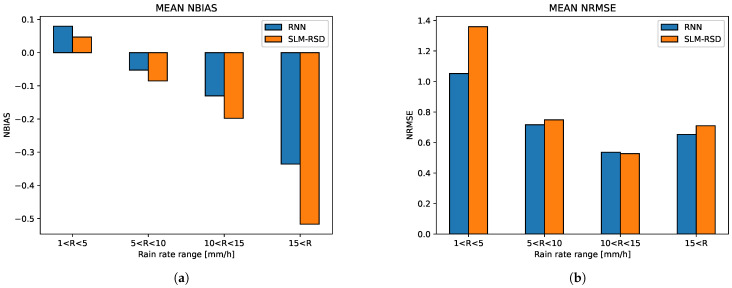
NBIAS and NRMSE results on the TEST dataset, averaged over links 1, 2, 4, 10, 15, 22, 26, 28, 29, 40, divided into different rain intensities. The blue bars represent the results for the RNN model and the orange bars represent the results for the SLM-RSD model. (**a**) NBIAS. (**b**) NRMSE. As can be seen, for events stronger than 5 mm/h, the difference in the RMSE between the two methods is small.

The performances of the models in terms of the NBIAS and NRMSE for different values of rainfall intensity are presented in Figure 6a,b on the TEST dataset, where the performance was averaged over links 1, 2, 4, 10, 15, 22, 26, 28, 29 and 40. In both figures, the blue bars represent the results for the RNN model, and the orange bars represent the results for the SLM-RSD model.

The RNN model achieves lower RMSE than the SLM-RSD model for all ranges of rainfall intensity. The major improvement in the RNN method is achieved at low rainfall intensities. For rain rates larger than 5 mm/h, the RNN method performs slightly better. Estimating light rainfall is obviously a harder task than estimating strong rainfall since the range in the attenuation values are smaller and closer to the quantization noise of the RSL signal. Therefore, the RNN model outperforms the short links model for light rainfall as expected.

**Figure 7:**
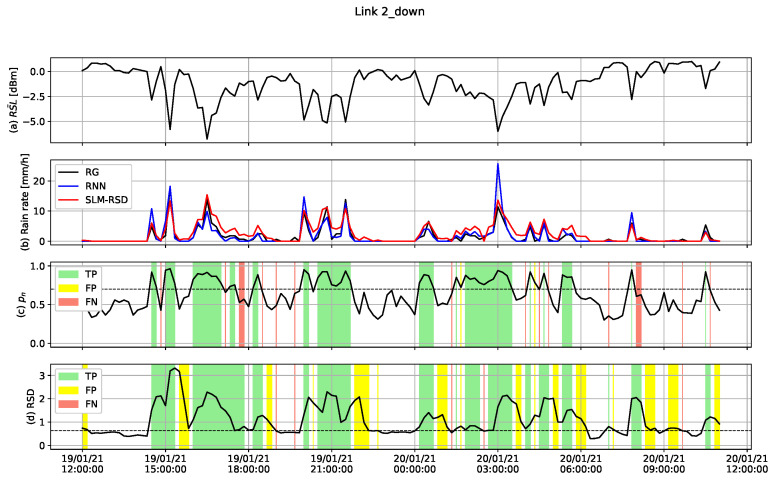
Rainfall classification and estimation results for link 2 from TEST dataset. (**a**) Normalized RSL. (**b**) Rain rate. In black: measured by the rain gauge, in blue: estimated from the RNN, in red: estimated from the SLM-RSD model. (**c**) $p_{n}$, the probability that the sample is considered wet from the RNN. (**d**) Rolling standard deviation of the RSD method for classification. In panels (**c**,**d**), TP samples were marked by green, FP samples were marked by yellow and FN samples were marked by red. A dashed line is plotted, showing the threshold used to determine wet or dry.

An example of the combined wet/dry classification and rainfall estimation results on the TEST dataset for link 2 is shown in Figure 7, with the corresponding rain-gauge measurements. The first panel from the top shows the normalized RSL signal obtained from link 2. In the second panel, the blue and red lines represent the rainfall estimation obtained by the RNN model and the SLM-RSD model, respectively. The black line represents the rain-gauge measurements. In the third panel, the wet/dry probability signal, $p_{n}$, of the RNN model is shown, and in the fourth panel, the RSD signal obtained from the RSL is shown. In both the third and fourth panels, a dashed line is plotted at the threshold level used to determine wet or dry. Moreover, samples that were classified correctly as wet (TP) were marked by green color. Samples that were classified incorrectly as wet (FP) were marked by yellow color, and samples that were classified incorrectly as dry (FN) were marked by red color. It can be seen that the RSD method results in a higher number of FP samples compared to RNN, which causes an estimation in rain rate larger than zero.

### 4.2. Rainfall Maps Comparison

In this subsection, we construct high-resolution 2D maps of 24 h-accumulated rainfall and compare the results to the rain-gauge-adjusted weather radar maps of the Israeli Meteorological Services (IMSs).

We apply inverse distance weighting (IDW) interpolation, as described in [19], to construct the rainfall maps, using the accumulated rainfall estimates from each link in the network.

(24) $$R\left(x\right)=\frac{\sum_{i=1}^{N_{L}}w_{i}\left(x\right){\hat{R}}^{i}}{\sum_{i=1}^{N_{L}}w_{i}\left(x\right)}$$

where:

(25) $$w_{i}\left(x\right)=\left\{\begin{array}{cc} \frac{{\left(1-\frac{d_{i}\left(x\right)}{D}\right)}^{2}}{{\left(\frac{d_{i}\left(x\right)}{D}\right)}^{2}} & \mathrm{if}\;\frac{d_{i}\left(x\right)}{D}\leq 1\\ 0 & \mathrm{if}\;\frac{d_{i}\left(x\right)}{D}>1 \end{array}\right.$$

R(x), in mm, is the interpolated rain at target grid point x; ${\hat{R}}^{i}$, in mm, is the estimated rain at the center of link *i*, xi, after the calibration process; $d_{i}\left(x\right)=||x-x_{i}{\left|\right|}_{2}$, in km, is the distance between the target grid point x and the center of link *i*; *D*, in km, is the radius of influence, i.e., the distance beyond which a link ceases to effect target point x; and $N_{L}$ is the number of links. The choice of the radius of influence should depend on the spatial autocorrelation function of the rain field and on the spatial density of the links. A large radius reduces the dependency on noisy links and results in maps that cover wider areas. Too large a radius can result in averaging local variability of the rain field. Here, we empirically set D=2 km and the spacing between the grid points to be 300 m to achieve a map that is large enough to cover most of the area of Rehovot, but also maintain the variability in the rain field measured by dense and short links.

**Figure 8:**
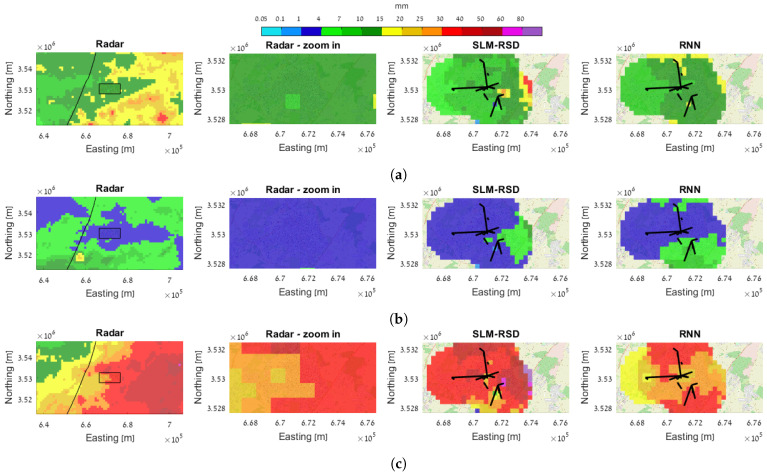
The 24 h-accumulated rainfall maps for three different events (each event is given in a single row). In each row from left to right: radar rainmap of the surrounding area; radar—cropped and zoomed to the dimensions of the estimated maps; SLM-RSD-based rainmap, and RNN-based rainmap. As can be seen, both estimation methods can be used to interpolate 2D rainmaps with good agreement with the radar-based map. (**a**) Event 1—14 January 2021 08:00 to 15 January 2021 08:00. (**b**) Event 2—18 January 2021 08:00 to 19 January 2021 08:00. (**c**) Event 3—19 January 2021 08:00 to 20 January 2021 08:00.

The 24 h-accumulated rainfall maps obtained from both methods are compared to the IMS-adjusted weather radar in Figure 8, where a single color bar is used across all events. Figure 8a–c are examples of different rain events from 2021, where the first is from a medium rainfall event, starting at 08:00 on 14 January; the second is from a light rainfall event, starting at 08:00 on 18 January; and the third is from a strong rainfall event, starting at 08:00 on 19 January. The first image from the left contains the IMS-adjusted radar map in a zoomed-out view of Rehovot and its surroundings. The second image is a zoomed-in view of the radar map of Rehovot’s area. The third image is the estimated rainfall map obtained by the SLM-RSD model, and the fourth image is the estimated rainfall map obtained by the RNN model.

In most cases, the estimated rain maps show an agreement with the average rainfall depth of the adjusted radar, indicating that the method is able to distinguish between light and strong rainfalls.

A comparison between the estimated and the adjusted radar maps using BIAS and RMSE was made. The metrics are calculated over the locations where the estimated values are available, i.e., up to 2 km distance from the center of the closest link. Table 6 summarizes the comparison results for the 24 h-accumulated rain maps.

Table 6 indicates that the bias is negative for strong rainfall events accumulated over 24 h, in accordance with Figure 6a, which shows that the rainfall estimation from the links underestimate the rain rate measured by the rain gauge at high rain intensities. In both cases, the RMSE is higher for stronger rainfall events, as expected. A low spatial correlation between the estimated and the adjusted radar map can be obtained in both cases. The high variability in the estimated rain map can be caused by the fact that the links measure the rain at ground level, whereas the radar measures the rain at higher altitude. Errors due to the quantization of the RSL, baseline determination and wet/dry classification also contribute to the differences between the maps.

## 5. Conclusions

In this paper, we conducted a comparison between two methods of rain estimation from RSL measurements of an existing smart-city network operating at E-band frequencies. The first method is based on a calibration process, where every link is assigned two parameters that are estimated from attenuation measurements of a reference link in the network. The second method is based on an RNN model, where we trained a set of links to detect wet and dry periods and estimate the rainfall intensity based on rain-gauge measurements, which serve as the ground truth.

When looking at the rain estimation results for a given link, the RNN-based network provided better results in terms of RMSE compared to the short links model, but the improvement itself was less than 20% in average performance, and the largest difference was achieved at lower rainfall intensities. This indicates that a simple (and almost) linear model can be used for medium to strong rainfalls.

A comparison of the estimated rain maps with the IMS radar maps when accumulated over 24 h resulted in low spatial correlation for both methods. However, the estimated maps show an agreement with the average intensity of the radar, and the two methods are able to distinguish between light and strong rainfalls, as well as produce maps with higher spatial resolution (with respect to the radar). Differences between the estimated maps and the radar maps can arise from changes in the rainfall intensity at different altitudes. The links measure the rainfall at street level, whereas the radar measures the rain at much higher altitude. In addition, the quantization of the RSL and errors in the baseline determination and the wet/dry classification also contribute to the differences between the maps.

Filtering out problematic links is a crucial step before constructing the rainfall maps. Noisy links with large fluctuations in the RSL can yield overestimation. The overestimation is prominent when the rainfall is accumulated for long periods, and this is directly related to the performance of the wet/dry classifier. The change in the RSL properties of some links between different years resulted in poor performance of both the short links model and the RNN. Therefore, a more frequent estimation of the model parameters should be performed.

## Figures and Tables

**Table 1:** Dataset duration and number of samples from each link.

Dataset	Duration in Hours	Samples
TRAIN	423	51 k
VALIDATION	111	13 k
TEST	240	29 k

**Table 2:** Datasets: wet vs. dry samples.

Dataset	Wet Ratio (%)	Dry Ratio (%)
TRAIN	25.59	74.41
VALIDATION	16.67	83.33
TEST	22.78	77.22

**Table 3:** Rain head configurations. Each FC layer contains RH input size neurons.

Model	RH Input Size	Architecture
Small RH	256	FC
Large RH	256	FC→ReLU→FC→ReLU→FC
Small RH plus Skip	278	FC
Large RH plus Skip	278	FC→ReLU→FC→ReLU→FC

**Table 4:** Model comparison on the TEST dataset based on links 1, 2, 4, 10, 15, 22, 26, 28, 29, 40.

Model	BIAS	RMSE	THR	ACC	Balanced ACC	Recall	Precision	F1
RNN—Small RH	−0.511	3.170	0.75	0.882	0.811	0.679	0.775	0.724
RNN—Large RH	0.587	3.986	0.73	0.873	0.837	0.770	0.702	0.734
RNN—Small RH plus Skip	−0.307	3.265	0.75	0.882	0.796	0.639	0.801	0.711
RNN—Large RH plus Skip	−0.134	3.381	0.77	0.880	0.783	0.605	0.819	0.696
SLM-RSD	−0.232	3.742	-	0.798	0.775	0.732	0.542	0.623

**Table 5:** Rainfall estimation results for each link.

Link ID	Length (km)	BIAS (mm/h)		RMSE (mm/h)		Corr.	
		SLM-RSD	RNN	SLM-RSD	RNN	SLM-RSD	RNN
1	1.7	−0.028	0.074	0.904	1.044	0.916	0.899
2	0.349	−0.005	−0.022	0.914	1.127	0.912	0.888
4	0.355	−0.143	−0.033	1.162	1.024	0.858	0.893
10	0.179	−0.174	−0.017	1.582	1.455	0.707	0.773
15	0.177	−0.188	−0.21	2.023	1.819	0.459	0.583
22	0.227	−0.234	0.152	1.639	2.052	0.683	0.681
26	0.563	0.782	−0.056	2.709	1.575	0.72	0.736
28	0.462	0.273	0.013	3.244	1.752	0.579	0.717
29	2.42	0.205	0.1	1.658	1.511	0.818	0.79
40	0.345	−0.312	−0.132	1.668	1.763	0.678	0.661

**Table 6:** Rain map comparison for 24 h accumulation time.

Start Date	End Date	BIAS (mm)		RMSE (mm)		RG (mm)	Spatial Average (mm)		
		SLM-RSD	RNN	SLM-RSD	RNN		Radar	SLM-RSD	RNN
14 January 2021 08:00	15 January 2021 08:00	−1.81	−0.49	4.75	2.94	14.00	11.83	10.02	11.34
18 January 2021 08:00	19 January 2021 08:00	0.25	1.40	1.73	2.09	2.10	2.70	2.95	4.10
19 January 2021 08:00	20 January 2021 08:00	7.39	−2.23	12.59	6.43	34.70	30.73	38.12	28.50
16 February 2021 08:00	17 February 2021 08:00	−4.17	−6.01	6.67	7.15	21.70	25.74	21.57	19.73
17 February 2021 08:00	18 February 2021 08:00	−10.50	−7.88	12.45	8.71	24.80	32.10	21.60	24.23
18 February 2021 08:00	19 February 2021 08:00	−0.52	−6.49	6.96	8.30	26.70	30.13	29.61	23.64
19 February 2021 08:00	20 February 2021 08:00	−2.92	−7.19	5.26	8.88	22.90	20.12	17.21	12.93

## Data Availability

The rainfall rain gauge measurements are available on http://www.meteo-tech.co.il/faculty/faculty_periodical.asp?client=1 (accessed on 11 April 2023) and provided by The Robert H Smith Faculty of Agriculture, Food and Environment (Rehovot), The Hebrew University of Jerusalem. The rainfall radar maps are available by request from the Israeli Meteorological Service (https://ims.gov.il/en (accessed on 11 April 2023)). The CMLs RSL measurements are proprietary data of SMBIT Ltd.
